# Stress and Mindfulness in Parkinson's Disease: Clinical Effects and Potential Underlying Mechanisms

**DOI:** 10.1002/mds.28345

**Published:** 2020-10-23

**Authors:** Anouk van der Heide, Marjan J. Meinders, Anne E.M. Speckens, Tessa F. Peerbolte, Bastiaan R. Bloem, Rick C. Helmich

**Affiliations:** ^1^ Department of Neurology, Centre of Expertise for Parkinson & Movement Disorders Radboud University Medical Centre Nijmegen the Netherlands; ^2^ Donders Institute for Brain, Cognition, and Behavior, Centre for Cognitive Neuroimaging Radboud University Nijmegen Nijmegen the Netherlands; ^3^ Radboud Institute for Health Sciences Radboud University Medical Centre Nijmegen the Netherlands; ^4^ Radboud University Medical Centre Department of Psychiatry, Centre for Mindfulness Nijmegen the Netherlands

**Keywords:** Parkinson's disease, mindfulness, psychological stress, anxiety, depression, quality of life

## Abstract

Patients with Parkinson's disease (PD) are very vulnerable to the negative effects of psychological distress: neuropsychiatric symptoms, such as anxiety and depression, are highly prevalent in PD; motor symptoms (such as tremor) typically worsen in stressful situations; and dopaminergic medication is less effective. Furthermore, animal studies of PD suggest that chronic stress may accelerate disease progression. Adequate self‐management strategies are therefore essential to reduce the detrimental effects of chronic stress on PD. Mindfulness‐based interventions encourage individuals to independently self‐manage and adapt to the challenges created by their condition. In PD, emerging clinical evidence suggests that mindfulness‐based interventions may reduce psychological distress and improve clinical symptoms, but insight into the underlying mechanisms is lacking. In this viewpoint, we provide a systematic overview of existing mindfulness trials in PD. Furthermore, we discuss the cerebral mechanisms involved in acute and chronic stress, and the impact of mindfulness‐based interventions on these networks. In addition, we delineate a hypothetical mechanistic framework of how chronic stress may increase the susceptibility for neuropsychiatric symptoms in PD and may potentially even influence disease progression. We end with offering recommendations for future research. © 2020 The Authors. *Movement Disorders* published by Wiley Periodicals LLC on behalf of International Parkinson and Movement Disorder Society

Parkinson's disease (PD) is the fastest growing neurological disorder in the world: the number of people with PD has doubled to more than 6 million in the last two decades. PD is characterized by the motor symptoms bradykinesia, rigidity, and tremor, but patients invariably also have nonmotor symptoms, such as anxiety, depression, cognitive impairment, sleeping problems, or constipation. The pathophysiological hallmark of PD is a profound loss of nigrostriatal dopaminergic neurons and, to a lesser extent, of serotoninergic and noradrenergic neurons. There is converging clinical evidence that patients with PD are very sensitive to the effects of psychological stress. First, the prevalence of stress‐related neuropsychiatric symptoms in PD is high: 30% to 40% for depression^1^ and 25% to 30% for anxiety.^2^ Second, widespread clinical evidence suggests that stressful episodes worsen certain PD motor symptoms, such as tremor,^3^ freezing of gait,^4^ and dyskinesia.^5^ Furthermore, we have recently shown in 358 patients with PD that coronavirus disease (COVID)‐related stress was associated with increased (self‐reported) severity of not only motor and neuropsychiatric symptoms (anxiety and depression), but also of nonmotor symptoms, such as pain, constipation, and sleeping difficulties.^6^ Third, dopaminergic medication can be less effective in reducing motor symptoms during stress, as has been shown for tremor.^7^ The increased sensitivity of patients with PD to stress may be related to dopaminergic dysfunction of the striatum: dopamine‐dependent adaptation (or flexibility) is a requirement for successful coping that, when deficient, leads to a sense of loss of control and increased psychological distress.^8^ In this viewpoint, we discuss the detrimental effect of chronic stress on PD and how mindfulness‐based interventions may mitigate this effect, both from a clinical and a mechanistic standpoint.

In healthy individuals, the physiological stress response is critical to anticipate real or perceived threats, to restore homeostatic balance.^9^ It consists of the fast adrenomedullary response that triggers (nor)adrenaline release, resulting in pupil dilation and increased heart rate, respiration and perfusion of active tissues, and the slower hypothalamic–pituitary–adrenal (HPA) axis stimulating cortisol secretion. The HPA system is modulated by negative‐feedback loops to protect against prolonged activity. However, chronic stress leads to dysregulation of this feedback mechanism, resulting in elevated glucocorticoid levels, and this has indeed been observed in PD cohorts.^10^ To reduce the detrimental effects of chronic stress in PD, adequate self‐management strategies are essential. In recent years, evidence for the effect of nonpharmacological treatments for PD, such as exercise, has accumulated,^11^ but the evidence for stress‐alleviating interventions is much less clear.

Mindfulness‐based interventions have at its core the encouragement of individuals to independently self‐manage and adapt to the challenges created by their condition. It is the trainable capacity to experience the present moment, on purpose and without judgment, while being resilient to experienced joy and sadness.^12^ The originally Buddhist tradition was used to develop a structured mindfulness‐based stress reduction course and later mindfulness‐based cognitive therapy. The key difference between these two is that mindfulness‐based cognitive therapy includes some cognitive behavioral techniques, such as relapse prevention strategies, in addition to the meditation exercises. Both interventions consist of an 8‐week structure where mediation exercises such as sitting and yoga exercises are alternated with dialogue and psychoeducation. In several chronic conditions, such as depression, cancer, chronic pain, and cardiovascular disease, positive effects of mindfulness‐based interventions have been reported on stress, anxiety, depression, physical functioning, and quality of life (QoL).^13^, ^14^ Mindfulness may have similar beneficial effects in PD.^12^ Here, we start by giving a systematic overview of previous mindfulness trials in PD, focusing on the effects on nonmotor (depression and anxiety) and motor symptoms and QoL. Then we discuss the cerebral networks involved in mediating stress and how mindfulness may impact on these networks. Next, we discuss the potential neurobiological mechanisms by which chronic stress may increase susceptibility for depressive and anxiety disorders and influence disease progression in PD. We end with offering recommendations for future research.

## Clinical Effect of Mindfulness‐Based Interventions

Nine studies tested the effects of a mindfulness‐based intervention on clinical symptoms in PD, and all studies reported positive results (Table 1). An overview of the search strategy, study characteristics, and quality assessment can be found in the Supporting Information. One study reported only qualitative findings; here we focused on the remaining eight quantitative studies. Interestingly, 6/8 trials reported a reduced depression score after mindfulness‐based interventions, and 4/7 studies reported reduced anxiety scores. Motor symptoms were assessed in only three studies, of which two reported significant improvement after a mindfulness‐based intervention. Two of eight studies found significant improvement in QoL.

**TABLE 1:** Reported change in reviewed articles after mindfulness‐based intervention

Study	Sample Size		Motor Symptoms Instrument (Maximum Score) Absolute Change (SD)		Depression Instrument (Maximum Score) Absolute Change (SD)		Anxiety Instrument (Maximum Score) Absolute Change (SD)		Quality‐of‐Life Instrument (Maximum Score) Absolute Change (SD)	
	Intervention	Control	Intervention	Control	Intervention	Control	Intervention	Control	Intervention	Control
Advocat (2016) [22]	n = 24	n = 33			DASS‐D (42)		DASS‐A (42)		PDQ‐39 (156)	
					*+1.9* ^a^	*+1.1*	*+0.3*	**−0.6**	**−0.5**	**−1.5**
Birtwell (2017) [23]	n = 6 (uncontrolled)				DASS‐D (42)		DASS‐A (42)		PDQ‐39 (156)	
					**−9.0** ^a^		**−7.5** ^a^		N.I.	
Cash (2016) [24]	n = 39 (combined: 29 patients with PD with 10 caregivers)				PHQ‐9 (27)		GAD‐7 (21)		PDQ‐39 (156)	
					**−1.6** ^a^		**−0.9**		**−2.4**	
Dissanayaka (2016) [12]	n = 14 (uncontrolled)		MDS UPDRS‐III (76)		HAM‐D (52)		GAI (20)		PDQ‐39 (156)	
			**−0.8**		**−0.8** ^a^		**−1.9** ^a^		**−2.8**	
Kwok (2019) [23]	n = 71	n = 67	MDS UPDRS‐III (76)^c^		HADS‐D (21)^c^		HADS‐A (21)^c^		PDQ‐8 (32)^c^	
			**−13.8** ^a^	**−9.1** ^a^	**−2.6**	**−0.3**	**−2.4**	**−0.4**	**−2.2**	*+0.5*
Pickut (2015) [25]	n = 14	n = 13	MDS UPDRS‐III (76)^c^		BDI				PDQ‐pain (12)	
			**−5.5** ^a^	*+1.1*	N.I.	N.I.			*+0.8* ^a^	**−0.7**
Rodgers (2019) [26]	n = 15	n = 12			DASS‐D (42)^c^		DASS‐A (42)^a^		PDQ‐39 (156)^a^	
					**−0.8** ^a^	*+0.4*	**−0.7**	**−1.3**	**−1.8**	**−3.0**
Son (2018) [24]	n = 33	n = 30			GDS (30)^b^		STAI (160)^b^		PDQL (185)^b^	
					**−3.4**	**−1.0**	**−6.5**	*+9.4*	**+17.4**	*−8.6*

Absolute changes between baseline and postintervention for measures relating to motor symptoms, depression, anxiety, and quality of life are listed. Values in parentheses are the maximum score per measure. Characteristics of studies and used measures can be found in the Supporting Information. Boldface indicates improvement; italics indicates worsening at postintervention.

^a^ Change (*P* < 0.05) between T1 (baseline) and T2 (postintervention).

^b^ Difference (*P* < 0.05) between groups (in controlled studies) at T2 (postintervention).

^c^ 2×2 interactions between time (preintervention and postintervention) and group (intervention and control).

Abbreviations: MDS UPDRS‐III, Movement Disorder Society Unified Parkinson's Disease Rating Scale Part III; BDI, Beck Depression Inventory; N.I., not indicated; DASS, Depression Anxiety Stress Scale (A = anxiety sub‐scale, D = depression sub‐scale); GAI, geriatric anxiety inventory; GAD, generalized anxiety disorder; GDS, geriatric depression scale; HADS, hospital anxiety and depression scale (A = anxiety sub‐scale, D = depression sub‐scale); HAM, Hamilton depression rating scale; PDQ, Parkinson's disease questionnaire; PDQL, Parkinson's disease quality of life; PHQ, patient health questionnaire; STAI, state‐trait anxiety inventory.

An important issue is whether these findings are clinically relevant. This is the case when a change is larger than the minimal clinically important difference (MCID), the smallest difference in score that informed patients perceive as important. With regard to anxiety and depression rating scales, MCIDs in PD populations have only been established for the Beck Depression Inventory,^15^ Geriatric Depression Scale (GDS)‐30,^16^ and Hospital Anxiety and Depression Scale (HADS)^17^ (Supporting Information). From this we can conclude that the improvement in HADS‐D depression score of 2.6 points and in HADS‐A anxiety score of 2.4 points reported by Kwok et al.^18^ is likely clinically relevant (HADS‐D: MCID = 1.7 points; HADS‐A: MCID = 1.8 points), whereas the improvement in GDS‐30 depression of 3.4 points reported by Son et al.^19^ is not (MCID = 5.4). For motor symptoms, all trials used Movement Disorder Society Unified Parkinson's Disease Rating Scale Part III (MDS‐UPDRS III) scores as an outcome measure, for which the MCID threshold was estimated to be 3.25 points.^20^ Only one of three studies showed a significant improvement of 13.8 points in the intervention group. For QoL questionnaires, the estimated MCID in patients with PD for the Parkinson's Disease Questionnaire (PDQ)‐39 is −4.7 points (improvement) and +4.2 points (worsening), whereas the MCID for the PDQ‐8 is −5.9 and +4.9 points.^21^ None of the included studies using the PDQ found a change that exceeded this threshold (Table 1). Taken together, for many studies it remains unclear whether the effects were clinically meaningful, either because the MCID for the outcome measure was unknown or because the reported effects did not exceed the MCID. Future studies may take this into account when choosing the primary outcome measure, using the MCID as the basis for a power analysis.

The largest randomized controlled trial yet in PD was performed in Hongkong and compared 71 patients who received mindfulness yoga training with 67 patients who received stretching and resistance exercises.^18^ This study reported that the mindfulness yoga intervention significantly improved depression, anxiety, motor scores, and QoL scores, as compared with the active control (Table 1). The data also show a remarkable improvement in the MDS‐UPDRS III. Average scores were reduced from 34.9 (SD 14.9) at study onset to 21.1 after the mindfulness yoga intervention (large effect size: Cohen's *d* = 0.93). It is noteworthy that these large motor improvements were not accompanied by clinically relevant improvements in QoL. This MDS‐UPDRS improvement is very large even when compared with other effective interventions. For example, in patients with PD starting with 100/25 mg levodopa/carbidopa 3x daily, total MDS‐UPDRS improved from 28.0 (SD 11.2) to 23.5 points (medium effect size: Cohen's *d* = 0.40).^27^ Therefore, although the data are encouraging, these findings must be replicated in future studies, which should also make clear whether results from an Asian population can be extrapolated to a Caucasian population. Taken together, previous studies suggest that mindfulness‐based interventions may improve depression and anxiety in PD, whereas the evidence for improved motor symptoms and QoL is less strong.

## Cerebral Effects of Stress and Mindfulness

**FIG. 1:**
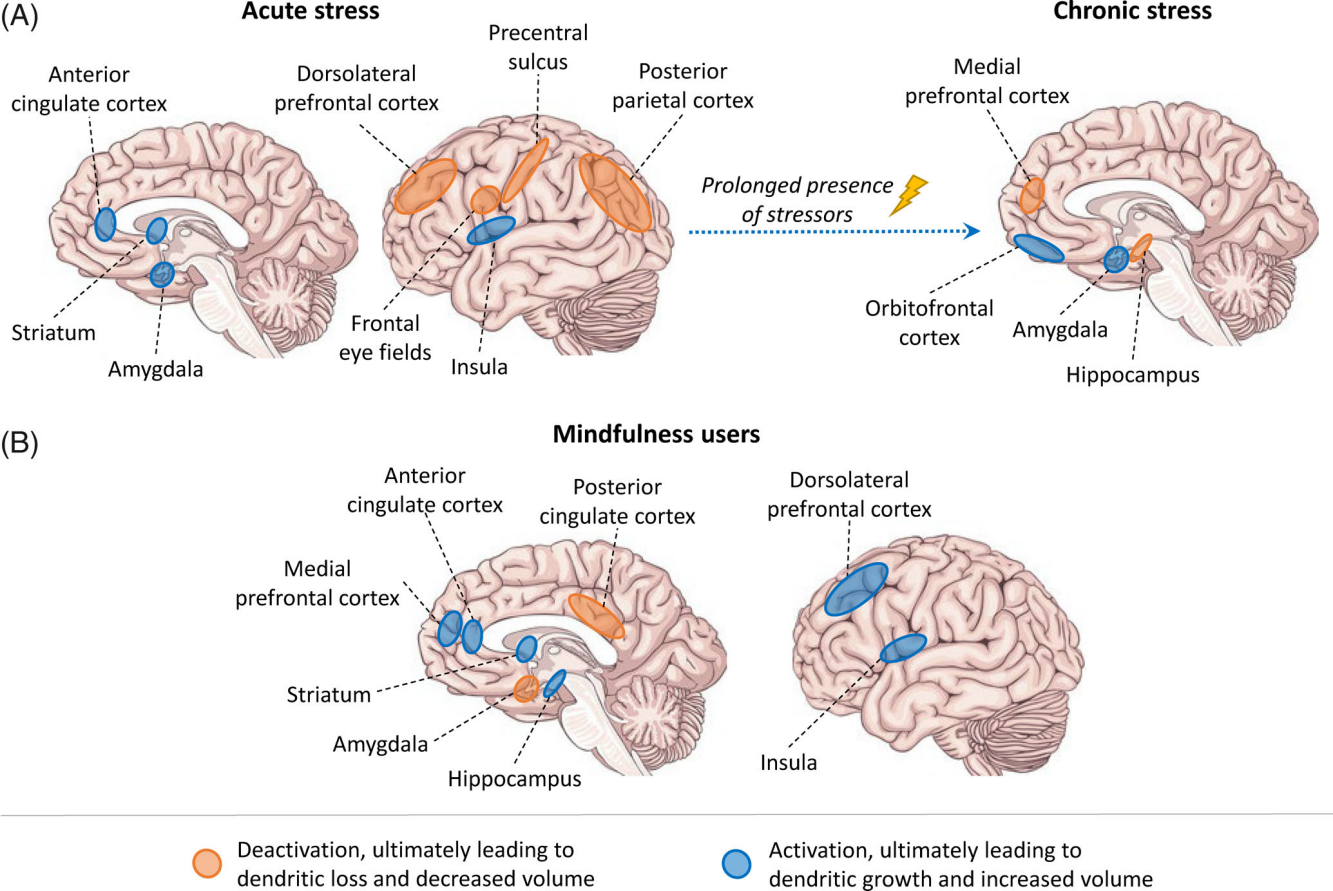
Brain network activity related to stress and mindfulness. (**A**) Brain regions are activated (blue) or deactivated (orange) during acute stress,^28^ resulting in neuroplastic structural changes in core regions after chronic stress.^29^ (**B**) Brain regions for which altered activation,^30^ as well as structural brain changes,^31^ have repeatedly been demonstrated in mindfulness practitioners. Blue regions consistently show activation during mindfulness‐related tasks or resting state, whereas orange regions were found to be deactivated. [Color figure can be viewed at wileyonlinelibrary.com]

Acute stress is associated with increased salience network activation, consisting of the amygdala, anterior cingulate, and insula.^28^ It also prompts deactivations in the central executive network, including the dorsolateral prefrontal cortex, posterior parietal cortex, precentral sulcus, and frontal eye fields. Chronic stress leads to neuroplastic changes in key nodes of these networks: growth of amygdala and orbitofrontal cortex, but shrinkage of the hippocampus and medial prefrontal cortex (Fig. 1A).^29^ After a mindfulness‐based intervention, reduced stress correlated with gray matter density decrease in the amygdala but increase in the hippocampus. In addition, mindfulness‐based intervention studies consistently showed increased activity,^30^ as well as structural changes,^31^ in the insula, anterior and posterior cingulate cortex, striatum, and the medial and dorsolateral prefrontal cortex (Fig. 1B). All of these regions have important roles in attentional control, emotional regulation, and self‐awareness, and they largely overlap with regions where activity changes during acute stress. We acknowledge that many other brain areas are likely also involved in mindfulness practice, but this requires more research. Only one trial investigated (structural) brain changes after a mindfulness‐based intervention in patients with PD.^32^ The intervention group showed increased gray matter density in the hippocampus, amygdala, caudate nucleus, left thalamus, temporoparietal junction, cuneus, left occipital lobe, and left parahippocampal gyrus. The usual care group showed decreased gray matter density in the cerebellum.

## Cerebral Effects of Chronic Stress in PD

**FIG. 2:**
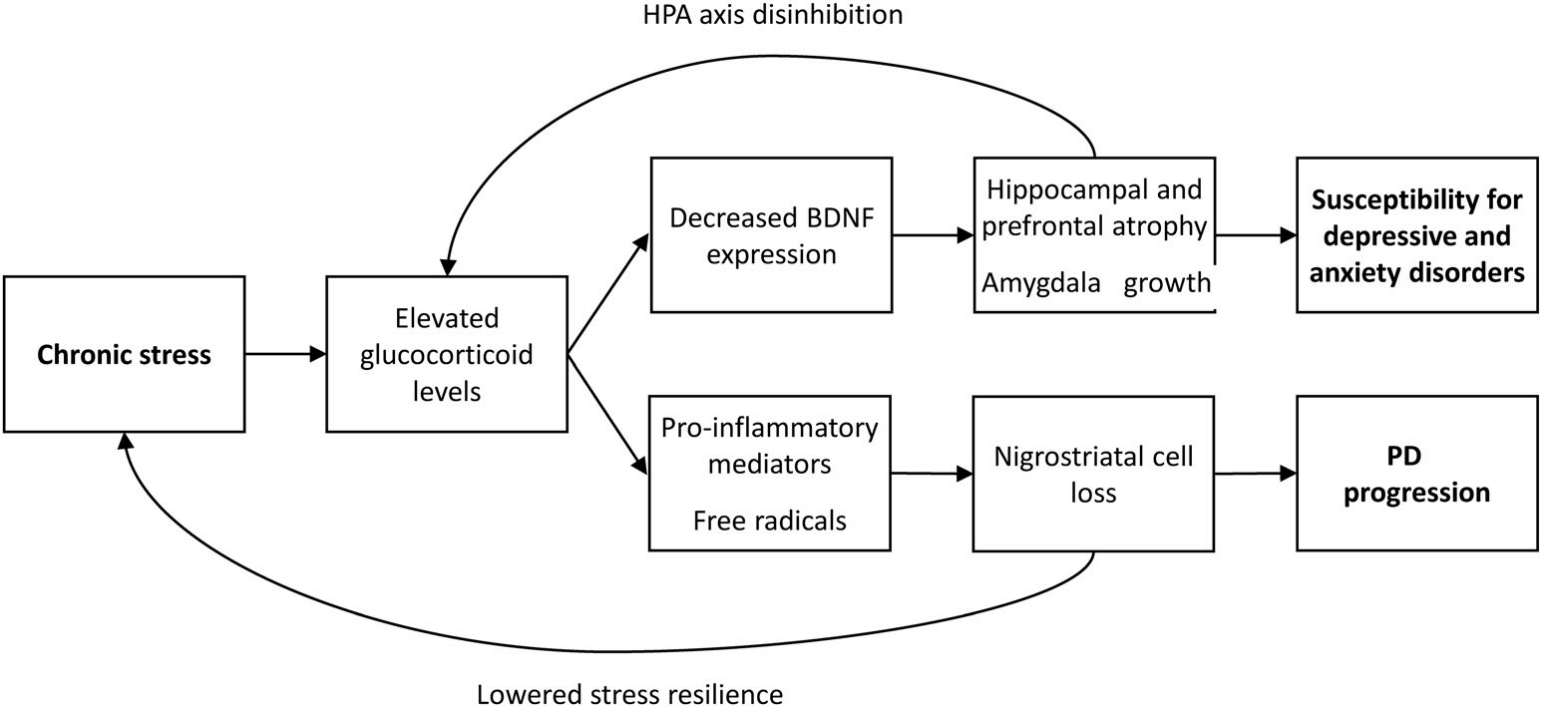
A pathophysiological model of chronic stress in Parkinson's disease (PD). This figure provides a hypothetical (and simplified) framework of how chronic stress in patients with PD may lead to higher susceptibility for depressive and anxiety disorders on the one hand, and to a more rapid progression of the disease on the other hand. Upper part: the high levels of glucocorticoids that result from chronic stress decrease the expression of brain‐derived neurotrophic factor (BDNF), which induces atrophy in the hippocampus and prefrontal cortex and growth in the amygdala.^33^ This increases the risk for development of depressive and anxiety disorders.^34^ Hippocampal atrophy also disinhibits the hypothalamic–pituitary–adrenal (HPA) axis, further increasing glucocorticoid levels.^35^ Lower part: elevated glucocorticoid levels also increase neuroinflammation^36^ and production of reactive oxygen species (ROS).^37^ These molecular changes may contribute to degeneration of nigrostriatal dopaminergic neurons.^38^

As outlined earlier, chronic stress influences the brain both at the systems level (large‐scale brain circuits) and at the molecular level (Fig. 2). Through these changes, chronic stress may affect PD brains by increasing the susceptibility to depressive and anxiety disorders, while also potentially impacting the already injured dopaminergic nigrostriatal system in patients with PD. Although this remains highly speculative at this stage, the following mechanisms may contribute to these effects.

First, high levels of glucocorticoids that result from chronic stress decrease the expression of brain‐derived neurotrophic factor, which facilitates stress‐induced remodeling of the hippocampus, amygdala, and prefrontal cortex.^33^ Several studies observed an increase in brain‐derived neurotrophic factor plasma level after mindfulness or meditation interventions,^39^ suggesting that mindfulness‐based interventions might counteract this stress‐induced remodeling. This stress‐induced atrophy in the hippocampus and prefrontal cortex and growth in the amygdala likely contribute to impaired cognitive function and emotion regulation, increasing the risk for development of depressive and anxiety disorders.^34^ Because of the inhibitory control of the hippocampus over the HPA axis,^40^ hippocampal atrophy also results in disinhibition of the HPA axis, causing a positive feedforward cascade that further increases glucocorticoid levels,^35^ which is another potential vicious circle. In a similar way, noradrenaline release during acute stress may influence processing in specific brain regions. For example, the noradrenaline system excites the thalamus during a cognitively demanding task, resulting in increased tremor power.^41^ Whether similar mechanisms apply during chronic stress is unclear.

Second, chronic stress might aggravate the progression of nigrostriatal cell loss in PD, accelerating disease progression. Findings in rodent models of PD have provided some evidence in that direction: chronic stress exacerbated dopaminergic and noradrenergic neuronal loss,^36^, ^42^ and injection of corticosterone (which is elevated in chronic stress) increased the severity of cerebral alpha‐synucleopathy.^43^ This might be driven by neuroinflammation: stress hormones (such as cortisol) affect major immune functions by increasing microglia activation,^36^ although inflammation can, of course, also be triggered by other factors. Interestingly, in subjects with mild cognitive impairment, mindfulness reduced levels of inflammatory biomarker C‐reactive protein (CRP).^44^ In PD, a meta‐analysis showed increased CRP levels, but it is unclear whether this is associated with stress.^45^ Furthermore, the increased energy demands needed to respond to chronic stress are associated with increased production of reactive oxygen species, resulting in oxidative damage in several areas, including cortical regions, hippocampus and striatum.^37^ These molecular changes may contribute to degeneration of nigrostriatal dopaminergic neurons.^38^ In PD, this could result in a vicious circle: dopaminergic dysfunction of the striatum increases the stress sensitivity of affected individuals (because of impaired coping and reduced behavioral flexibility), while resulting psychological distress could negatively impact nigrostriatal dysfunction.^8^ Additional research may test this hypothesis.

Another way in which stress might influence PD disease progression is by depleting compensatory mechanisms. In PD, clinical symptoms usually become apparent when >50% of dopaminergic cells are lost. This suggests that compensatory mechanisms must take place in the early phase of PD to prevent overt clinical symptoms. These compensatory mechanisms are thought to take place both in the striatal dopamine system and at the level of large‐scale brain networks.^46^ Under stressful conditions, the residual dopaminergic function is compromised, and attentional resources are depleted, in a similar way as dual tasking does.^47^ This may “unmask” clinical symptoms that were not seen before or increase the severity of already manifest symptoms.

## Recommendations for Future Studies

Future trials should be adequately powered to demonstrate a clinically meaningful difference between a mindfulness and control intervention (based on MCIDs). The optimal control intervention should have a similar duration and amount of personal contact as mindfulness and should not differ in degree of physical exercise.^48^ When investigating the effect of mindfulness on disease progression, it is possible that interventions longer than 8 weeks are needed (eg, 18 months in the Age‐Well study in elderly adults^49^). Promising outcome measures include (self‐reported) anxiety and depression (Table 1), and it might be considered to test the merits of mindfulness in a sample of patients with PD scoring high on these symptoms.^50^ It would also be informative to assess effects on other nonmotor symptoms, such as pain, sleeping problems, and digestive issues. Any effects on motor symptoms are currently less clear, and these may be better investigated using wearable devices to identify even subtle improvements (eg, to detect effects on stress‐sensitive symptoms such as tremor) or using functional outcome measures. Specifically, such functional measures could include brain imaging (magnetic resonance imaging), activity of the HPA axis (eg, hair cortisol^10^), or inflammatory markers (eg, CRP^45^), and these may help to better understand the underlying working mechanisms. Follow‐up should ideally be 6 months or longer, because the effects of a mindfulness‐based intervention may consolidate with longer follow‐up.^14^

## Author Roles

Anouk van der Heide: conception, execution, and drafting the manuscript; Marjan J. Meinders: review and critique; Anne E.M. Speckens: review and critique; Tessa F. Peerbolte: review and critique; Bastiaan R. Bloem: conception and review and critique; Rick C. Helmich: conception, execution, and drafting the manuscript.

## Financial disclosures

Anne E.M. Speckens receives funding from the Dutch Cancer Foundation and the Netherlands Organization for Scientific Research. Bastiaan R. Bloem receives funding from the Parkinson's Foundation, the Netherlands organization for Scientific Research, International Parkinson Fonds, and the Michael J. Fox Foundation. The Parkinson Center of the Radboud University Medical Center was supported by a center of excellence grant of the Parkinson's Foundation. Rick C. Helmich was supported by the Michael J. Foundation (grant #16048) and by the Netherlands Organization for Scientific Research (VENI grant #91617077). Rick C. Helmich serves on the Clinical Advisory Board of Cadent Therapeutics.

## Financial disclosures

Anne E.M. Speckens receives funding from the Dutch Cancer Foundation and the Netherlands Organization for Scientific Research. Bastiaan R. Bloem receives funding from the Parkinson's Foundation, the Netherlands organization for Scientific Research, International Parkinson Fonds, and the Michael J. Fox Foundation. The Parkinson Center of the Radboud University Medical Center was supported by a center of excellence grant of the Parkinson's Foundation. Rick C. Helmich was supported by the Michael J. Foundation (grant 16048) and by the Netherlands Organization for Scientific Research (VENI grant 91617077). Rick C. Helmich serves on the Clinical Advisory Board of Cadent Therapeutics.

## Supporting information


**Appendix S1** Supporting Information.
