# Supplementary material for: Stress and Mindfulness in Parkinson's Disease: Clinical Effects and Potential Underlying Mechanisms
Source: Mov Disord. 2020 Oct 23;36(1):64–70. doi: 10.1002/mds.28345 (PMC7894549; doi:10.1002/mds.28345)
Supplement: Supplementary file 1 — Appendix S1 Supporting Information. [file MDS-36-64-s001.docx]

***Supplement 1: Search strategy and study quality assessment***

*Search strategy and selection criteria*

We performed a computerized search for research‐based publications between 1950 and March 2020 in the electronic databases of PubMed, Medline, Embase, The Cochrane Library and Web of Science. In addition, we screened the reference lists from included articles. We cross‐referenced “Parkinson's disease” and synonyms with “mindfulness” within article titles and abstracts, and excluded conference abstracts in the search. Separate searches with alternative spellings were also included. Duplicates and articles that appeared irrelevant based on title and abstract screening were excluded. Studies were selected using the following inclusion criteria: articles in English from peer‐reviewed journals, a primary study population of patients with idiopathic PD and an applied mindfulness related intervention. We chose not to be very strict about which types of mindfulness interventions to include and allowed all interventions in which mindfulness clearly was the core skill taught. We included both randomized controlled trials (RCTs) and studies with no control group. These criteria were not used in the search phase but instead applied at the time of screening and sensitivity analysis.

After removal of duplicates, the search revealed a total of 55 articles. The process of the literature search and study selection is shown in Supplementary Figure 1 below. For every step in the selection process, we noted how many studies were discarded, and for what reason. In the end, this selection yielded nine studies that were suitable for analysis. Nine studies remained for data extraction and quality assessment: characteristics are provided in Supplementary Table 1. Interventions differed between studies, including mindfulness-based stress reduction (MBSR) (n=4), mindfulness-based cognitive therapy (MBCT) (n=3), mindfulness yoga (n=1) and mindfulness meditation-based complex exercise (n=1). The reported outcomes attributed to these mindfulness-based interventions included depression (n=8), anxiety (n=7), motor symptoms (n=3), and quality of life (n=8). Sample sizes ranged from 6 to 138 PD patients. Five studies were RCT and used a PD control group^1-5^, one study had an uncontrolled combined sample of PD patients and caregivers^6^ and three studies were uncontrolled^7-9^. One study used only qualitative outcome measures^8^, the other eight studies had both qualitative and quantitative outcome measures consisting of self-report rating scales and investigator-reported MDS-UPDRS III scores.

*Study quality assessment*

We analyzed the methodological quality of the included studies. For the studies that used a control group, quality was assessed using the Cochrane risk of bias tool for randomized trials^10^. For uncontrolled designs, we applied the ROBINS-I tool for non-randomized studies^11^. All five RCTs were assessed as having a high risk of bias. Specifically, two studies lacked complete information on randomization regarding concealment. Furthermore, four of the five controlled studies made use of a passive control group. Participants in such treatment as usual or waiting list control groups, who might have had a positive expectation of the effect of mindfulness, may be disappointed and report worse outcomes hence causing an overestimation of treatment effects. The remaining RCT had an active control group, but did not specify a primary outcome and did not report what happened between the intervention and follow-up^3^. Positive was that all studies clearly followed protocol and deviations from intended interventions were minimal. All studies reported following their prespecified plan, except for one study that did not report all their outcome measures^2^, which is problematic, since primary and secondary outcomes must be predetermined so that when reporting results, a distinction can be made between testing hypotheses and explorative analyses. The four non-randomized trials were all associated with a serious risk of bias as well. In all studies, interventions were well defined, but confounding was likely, since various domains were not controlled for. For example, one study included some participants into the study after the intervention had already taken place, which may result in selection bias^8^. Protocol deviations were present in one study, for example omitting walking meditation due to gait instability in participants, but there is no reason to assume that this influenced outcome^7^. One study did not report whether there were deviations^8^. In three of the four studies, data were relatively complete, although dropout rates were high, ranging from 18 to 54%. Crucially, one study did not report all major outcome measures^9^. All studies kept to an a priori plan and there was no indication of selection of the cohort for analysis or reporting.

Supplementary Figure 1: Literature review flow chart





The process of the literature search and study selection. The search was done in five databases, which resulted in a total of 55 studies. For every step in the selection process, we noted how many studies were discarded, and for what reason. This selection yielded nine studies that were suitable for analysis.

Supplementary Table 1: Characteristics of studies on mindfulness in Parkinson’s disease

| **Study** | **Number of participants** | | **Age, range in years, mean (SD)** | **Gender, % women** | **Type of intervention** | **Number of sessions** | **Session duration** | **Follow up after intervention** |
| --- | --- | --- | --- | --- | --- | --- | --- | --- |
| Advocat^1^ | N=57 | intervention N=24 | 46 – 74  62.8 (7.6) | 67% | MBSR | 6 | 2 h | 20 weeks |
|  |  | control N=33 | 39 – 75  63.7 (8.6) | 52% | Waitlist |  |  |  |
| Birtwell^9^ | N=6 | | 60 – 72  68.0 (5.6) | 17% | MBSR | 8 | 2 h | 8 weeks |
| Cash^6^ | N=39 | PD patients N=29 | 50 – 82  65.6 (7.6) | 46% | MBSR | 8 (+ silent retreat) | 1.5 h | None |
|  |  | Caregivers N=10 |  |  |  |  |  |  |
| Dissanayaka^7^ | N=14 | | 52 – 78  66 (7.4) | 36% | MBCT | 6 | 2 h | 20 weeks |
| Fitzpatrick^8^ | N=12 | | 60 – 72  66.3 (7.3) | 42% | MBCT | 8 | 2.5 h | None |
| Kwok^3^ | N=138 | intervention N=71 | 38 – 85  63.7 (8.7) | 48% | mindfulness yoga | 8 | 1.5 h | 12 weeks |
|  |  | control N=67 | 38 – 85  63.5 (9.3) | 58% | stretching and resistance exercises | 8 | 1 h |  |
| Pickut^2^ | N=27 | intervention N=14 | Range N.I.  61.4 (11.3) | 50% | MBSR | 8 | 2.5 h | None |
|  |  | control N=13 | Range N.I.  62.2 (6.6) | 46% | Usual care |  |  |  |
| Rodgers^5^ | N=27 | intervention N=15 | 40 – 77  63.7 (8.76) | 33% | MBCT | 6 | 2 h | None |
|  |  | control N=12 |  | 58% | Waitlist |  |  |  |
| Son^4^ | N=63 | intervention N=33 | N.I. | 58% | MMBCEP | 6 | 2 h | none |
|  |  | control N=30 | N.I. | 70% | Usual care |  |  |  |

The characteristics of the study population and the type and characteristics of the interventions presented by articles that we included in the review analysis. MBSR = mindfulness-based stress reduction, MBCT = mindfulness-based cognitive therapy, MMBCEP = mindfulness meditation-based complex exercise program. N.I. stands for ‘not indicated’.

***Supplement 2: Outcome measures and score options used in reviewed mindfulness trials***

|  | **Measure** | **No. of items** | **Max score** | **Response options** | **Clinical cutoff score and MCID** |
| --- | --- | --- | --- | --- | --- |
| DASS-D | Depression Anxiety Stress Scale (depression subscale) | 14 | 42 | 0 (not at all or ever applicable) – 3 (certainly or mostly applicable) | No PD clinical cutoff or MCID information found |
| PDQ-39 | Parkinson’s Disease Questionnaire-39 | 39 | 156 | 0 (never) – 4 (always) | Score is on 0-100% scale, MCID=4.2 in PD^12^ |
| PDQ-8 | Parkinson’s Disease Questionnaire-8 | 8 | 32 | 0 (never) – 4 (always) | Score is on a 0-100% scale  MCID=4.9 in PD^12^ |
| PHQ-9 | Patient Health Questionnaire-9 | 9 | 27 | 0 (not at all) – 3 (nearly every day) | Clinical cutoff ≥8/9^13^  No MCID for PD found |
| GAD-7 | Generalized Anxiety Disorder-7 | 7 | 21 | 0 (not at all) – 3 (almost every day) | No PD clinical cutoff or MCID information found |
| MDS UPDRS-III | Unified Parkinson's Disease Rating Scale part 3: Motor Examination | 19 | 76 | 0 (normal) – 4 (severe) | Scored by a physician, MCID=3.25 in PD^14^ |
| HAM-D | Hamilton Depression Rating Scale | 17 | 52 | 0 (normal) – 2 (severe) for 8 items, 0 (normal) – 4 (severe) for 9 items | Clinical cutoff in PD ≥9/10^13^  No MCID for PD found |
| GAI | Geriatric Anxiety Inventory | 20 | 20 | 0 (disagree) – 1 (agree) | Clinical cutoff ≥6/7 in PD^15^ |
| HADS-D | Hospital Anxiety and Depression Scale (depression subscale) | 7 | 21 | 0 (not at all) – 3 (almost always) * | Clinical cutoff ≥11 in PD^15^  MCID=1.72 in PD^16^ |
| DASS-A | Depression Anxiety Stress Scale (anxiety subscale) | 14 | 42 | 0 (not at all or ever applicable) – 3 (certainly or mostly applicable) | No PD clinical cutoff or MCID information found |
| HADS-A | Hospital Anxiety and Depression Scale (anxiety subscale) | 7 | 21 | 0 (not at all) – 3 (almost always) * | Clinical cutoff ≥10/11 in PD^16^  MCID=1.84 in PD^16^ |
| GDS-30 | Geriatric Depression Scale | 30 | 30 | 0 (no) – 1 (yes) | Clinical cutoff ≥9/10 in PD^13^  MCID=5.4 in PD^17^ |
| PDQL | Parkinson's Disease Quality of Life | 37 | 185 | 1 (permanently) – 5 (never) | No PD clinical cutoff or MCID information found |
| STAI | State-Trait Anxiety Inventory | 40 | 160 | 1 (not at all) – 4 (almost always) | No PD clinical cutoff or MCID information found |
| BDI | Beck’s Depression Inventory | 21 | 63 | 0 (no symptom impact) – 3  (maximum symptom impact) | Clinical cutoff ≥13/14 in PD^13^ MCID=3.3 in PD^18^ |

MCID=minimal clinical important difference. * These response options are used in most questions. The scale uses several response options that differ per question.

# ***References***

1. Advocat J, Enticott J, Vandenberg B, Hassed C, Hester J, Russell G. The effects of a mindfulness-based lifestyle program for adults with Parkinson's disease: a mixed methods, wait list controlled randomised control study. BMC Neurol 2016;16:166.

2. Pickut B, Vanneste S, Hirsch MA, et al. Mindfulness Training among Individuals with Parkinson's Disease: Neurobehavioral Effects. Parkinsons Dis 2015;2015:816404.

3. Kwok JYY, Kwan JCY, Auyeung M, et al. Effects of Mindfulness Yoga vs Stretching and Resistance Training Exercises on Anxiety and Depression for People With Parkinson Disease: A Randomized Clinical Trial. JAMA Neurol 2019;76:755-763.

4. Son HG, Choi E-O. The Effects of Mindfulness Meditation-based Complex Exercise Program on Motor and Non-Motor Symptoms, and Quality of Life in Patients with Parkinson's Disease. Asian Nurs Res 2018;12:145-153.

5. Rodgers SH, Schutze R, Gasson N, et al. Modified Mindfulness-Based Cognitive Therapy for Depressive Symptoms in Parkinson's Disease: a Pilot Trial. Behav Cogn Psychother 2019;47:446-461.

6. Cash TV, Ekouevi VS, Kilbourn C, Lageman SK. Pilot Study of a Mindfulness-Based Group Intervention for Individuals with Parkinson's Disease and Their Caregivers. Mindfulness 2016;7:361-371.

7. Dissanayaka NNW, Jion FI, Pachana NA, et al. Mindfulness for Motor and Nonmotor Dysfunctions in Parkinson's Disease. Parkinsons Dis 2016:7109052.

8. Fitzpatrick L, Simpson J, Smith A. A qualitative analysis of mindfulness-based cognitive therapy (MBCT) in Parkinson's disease. Psychol Psychother 2010;83:179-192.

9. Birtwell K, Dubrow-Marshall L, Dubrow-Marshall R, Duerden T, Dunn A. A mixed methods evaluation of a Mindfulness-Based Stress Reduction course for people with Parkinson's disease. Complement Ther Clin Pract 2017;29:220-228.

10. Higgins JP, Altman DG, Gøtzsche PC, et al. The Cochrane Collaboration’s tool for assessing risk of bias in randomised trials. BMJ 2011;343:d5928.

11. Sterne JA, Hernán MA, Reeves BC, et al. ROBINS-I: a tool for assessing risk of bias in non-randomised studies of interventions. BMJ 2016;355:i4919.

12. Horvath K, Aschermann Z, Kovacs M, et al. Changes in Quality of Life in Parkinson's Disease: How Large Must They Be to Be Relevant? Neuroepidemiology 2017;48:1-8.

13. Torbey E, Pachana NA, Dissanayaka NN. Depression rating scales in Parkinson's disease: A critical review updating recent literature. J Affect Disord 2015;184:216-224.

14. Horvath K, Aschermann Z, Acs P, et al. Minimal clinically important difference on the Motor Examination part of MDS-UPDRS. Parkinsonism Relat D 2015;21:1421-1426.

15. Dissanayaka NNW, Torbey E, Pachana NA. Anxiety rating scales in Parkinson's disease: a critical review updating recent literature. Int Psychogeriatr 2015;27:1777-1784.

16. Rodriguez-Blazquez C, Frades-Payo B, Forjaz MJ, de Pedro-Cuesta J, Martinez-Martin P, Longitudinal Parkinson's Disease Patient Study G. Psychometric attributes of the Hospital Anxiety and Depression Scale in Parkinson's disease. Mov Disord 2009;24:519-525.

17. Huang SL, Hsieh CL, Wu RM, Lu WS. Test-retest reliability and minimal detectable change of the Beck Depression Inventory and the Taiwan Geriatric Depression Scale in patients with Parkinson's disease. PLoS One 2017;12:e0184823.

18. Visser M, Leentjens AF, Marinus J, Stiggelbout AM, van Hilten JJ. Reliability and validity of the Beck depression inventory in patients with Parkinson's disease. Mov Disord 2006;21:668-672.
